# *Paramecium tetraurelia *basal body unit isolation for Cryo-electron tomography studies

**DOI:** 10.1186/2046-2530-4-S1-P68

**Published:** 2015-07-13

**Authors:** S Trépout, M Lemullois, P Guichard, F Koll, A Aubusson-Fleury, J Beisson, J Cohen, S Marco, AM Tassin

**Affiliations:** 1 Campus Universitaire, Bat 112, INSERM U759, Orsay Cedex, France; 2 Centre de Recherche, Institut Curie, Orsay F-91405, France; 3 UPR 3404-Centre de Génétique Moléculaire, Gif sur Yvette, CNRS, Gif Sur Yvette, Paris, France; 4 School of Life Sciences, Swiss Federal Institute of Technology (EPFL), Swiss Institute for Experimental Cancer Research (ISREC), Lausanne, Switzerland

## Objective

The Transition Zone (TZ) is defined as the most proximal region of the cilium overlapping with the most distal region of the basal body. This zone has been shown to play a crucial role in cilia biology since it is considered as the site of sorting of proteins that transit to cilia. Protein complexes housed at this zone are found mutated in MKS/NPHP ciliopathies. Although its organization varies from organism to organism, the TZ molecular composition and function are highly conserved. In *Paramecium*, the TZ is well structured with three distinct plates defined as the terminal, the intermediate and the axosomal plates. In this model, structural and molecular changes of the TZ are observed as anchored basal bodies become ciliated. Therefore, *Paramecium *appears to be a pertinent model to study the TZ at an ultrastructural level in correlation with its functionality.

## Methods

To reach this goal, we have developed a technique to isolate *Paramecium *basal body cortex units. These units fit the cryo-electron tomography requirements allowing us their visualisation in native conditions at nanometric resolution.

## Results

First cryo-tomograms obtained on these *Paramecium *units allow the observation of well-preserved basal bodies revealing the TZ with its three recognisable plates and the Y-links as well as at the proximal part of the basal body, the cartwheel and the radial spokes.

## Conclusion

Thus, studies of the consequences of TZ protein depletion at high resolution are now achievable by combining new isolation protocols and cryo-electron tomography.

